# Oral Health of Patients Treated with Acrylic Partial Dentures Using a Toothpaste Containing Bee Product

**DOI:** 10.1155/2017/4034179

**Published:** 2017-02-06

**Authors:** Karolina Wiatrak, Tadeusz Morawiec, Rafał Rój, Anna Mertas, Agnieszka Machorowska-Pieniążek, Patryk Kownacki, Marta Tanasiewicz, Małgorzata Skucha-Nowak, Stefan Baron, Tomasz Piekarz, Maciej Wrzoł, Mateusz Bogacz, Jacek Kasperski, Iwona Niedzielska

**Affiliations:** ^1^Department of Oral Surgery, School of Medicine with the Division of Dentistry in Zabrze, Medical University of Silesia in Katowice, Plac Akademicki 17, 41-902 Bytom, Poland; ^2^Department of Prosthetic Dentistry, School of Medicine with the Division of Dentistry in Zabrze, Medical University of Silesia in Katowice, Plac Akademicki 17, 41-902 Bytom, Poland; ^3^Department of Microbiology and Immunology, School of Medicine with the Division of Dentistry in Zabrze, Medical University of Silesia in Katowice, Jordana 19, 41-808 Zabrze, Poland; ^4^Department of Orthodontics, Chair of Masticatory Dysfunction and Orthodontics, School of Medicine with the Division of Dentistry in Zabrze, Medical University of Silesia in Katowice, Plac Traugutta 2, 41-800 Zabrze, Poland; ^5^Department of Conservative Dentistry with Endodontics, School of Medicine with the Division of Dentistry in Zabrze, Medical University of Silesia in Katowice, Plac Akademicki 17, 41-902 Bytom, Poland; ^6^Chair of Masticatory Dysfunction and Orthodontics, School of Medicine with the Division of Dentistry in Zabrze, Medical University of Silesia in Katowice, Plac Traugutta 2, 41-800 Zabrze, Poland; ^7^Department and Hospital of Craniomaxillofacial Surgery and Oral Surgery, School of Medicine with the Division of Dentistry in Zabrze, Medical University of Silesia in Katowice, Plac Akademicki 17, 41-902 Bytom, Poland

## Abstract

This study was carried out to investigate the influence of a propolis and tee tree oil-containing hygienic agent on selected oral health parameters, oral microflora, and the condition of periodontal health. Thirty-seven patients who underwent oral rehabilitation with a removable acrylic denture were selected and randomly assigned into two groups: study group (A) which received a newly formulated propolis and tee tree oil-containing toothpaste or a control group (C) without an active ingredient. API, S-OHI, and mSBI were assessed in three subsequent stages. During each examination swabs were employed for microbiological inoculation: in the study group after 4 weeks use of the active toothpaste showed a decrease in the number of isolated microorganisms. In the control group, after 4 weeks use of the toothpaste without active ingredients resulted in increase in the number of the isolated microorganisms. Improvements in hygiene and the condition of periodontium were observed in patients using active toothpastes. In the study group the oral flora diversity was reduced by the decrease in the number of cultured microorganism species, while in the control group an increase in the number of cultured microorganisms and their species was observed.

## 1. Introduction

Oral health and condition reflect body's general health. Condition of the oral cavity, level of hygiene, and residing pathogens affect the whole human organism. There are numerous reports on oral bacteria being the cause of bacterial endocarditis, pneumonia, gastric infections, chronic obstructive pneumonia, and other diseases [1]. The risk of contracting the above-mentioned diseases increases with age. Moreover, the amount of patients who use dentures, which are yet another place of accumulation of microorganisms associated with these infections, rises with age as well. Absences of teeth are closely related to both oral and general health [2]. Loss of a single tooth may cause rotation, shifting, and tipping of neighbouring teeth towards the empty space, which will in turn lead to adverse loads on these teeth, not along the long axis of the tooth [3–5]. Partial lack of teeth, especially in lateral teeth, may cause the loss of mandibular occlusal support, which leads to changes in its position in relation to the maxilla [6]. Such a shift in mandibular position may result in muscle fatigue and other perpetuated occlusal relations in temporomandibular joints [7, 8].

Prosthetic treatment is not only designed to restore partial lacks of teeth. It also serves a prophylactic purpose by preventing the formation of pathological changes in the stomatognathic system and removing already existing changes [9]. It is common to employ acrylic partial dentures. Such dentures shift the loads through mucous membrane and periosteum to the bone, restoring masticatory function to a limited extent, do not protect the residual dentition, cause periodontitis and mucositis, create favourable conditions for the accumulation of bacterial plaque, and accelerate the loss of alveolar process. Due to the adverse impact of partial dentures on the denture medium and residual dentition it is necessary to maintain proper oral hygiene, remove plaque carefully, and take preventive measures against caries and periodontopathy [10, 11]. Regardless of the type of prosthetic restoration chosen, the maintenance of proper denture and oral hygiene is indispensable to achieve a successful treatment outcome [12].

The oral cavity constitutes a natural environment for microflora, whose composition is an individual feature. It is influenced by, inter alia, age, topography of the oral cavity, condition of teeth, nutritional and breathing habits, tobacco smoking, oral hygiene, and loss of teeth or usage of prosthetic restorations [13]. The extent of such changes depends on the type of dentures: fixed dentures cause local changes in the microflora, while removable dentures cause more complex changes. It is associated with the formation of new reservoirs of microorganisms such as the intramucosal surface of the denture base or clasps on retaining teeth. Acrylic base of partial dentures covers a substantial surface of the mucosa, creating favourable conditions for the buildup of bacteria and fungi in the form of denture plaque. It is a place characterised by high humidity, elevated temperature, reduced oxygen supply, and impaired conditions for salivary self-cleaning. Moreover, the acrylic material, due to its heterogeneous and porous structure, absorbs water and swells in the oral cavity, further facilitating the aggregation of microorganisms [14–16]. Perfect oral hygiene is also the basic method for prevention of caries and diseases of the periodontium and oral mucosa. Caries and periodontal diseases are the most common cause of teeth loss. Therefore, it is crucial to protect residual dentition in patients with partial dentures from these diseases. The basic hygienic treatment is mechanical removal of plaque, supported by, for example, antimicrobial chemical agents [17, 18]. The positive effect of ethanolic extract of propolis and tea tree oil on the maintenance of proper oral hygiene has been known for a long time. They are naturally occurring compounds with a broad-spectrum antimicrobial, antiviral, antifungal, and antiprotozoal activity. Additionally, they exhibit antiphlogistic and antioxidant properties [19, 20].

Propolis, so-called bee glue, is made by bees mostly from resinous substances gathered from buds of some trees. Its properties depend on the species of bees which produce it as well as on the plants used for the production [21]. Propolis is a very complex substance with high contents of active ingredients like flavonoids, phenols, aromatic acids, esters, wax substances, aldehydes, coumarin, sterols, enzymes, or fatty acids, as well as proteins, microelements, and vitamins [22, 23]. Propolis is well-soluble in ethyl alcohol; thus medical extracts can easily be prepared [23]. In dentistry propolis is used in the treatment of caries [24] and periodontal diseases [20, 25]. It also exhibits analgesic activity and accelerates wound healing, which is used in dental surgery [26]. There are numerous scientific reports on its anticancer, antiphlogistic, and immunomodulating properties [19, 27]. Tea tree oil is obtained from leaves of a tree growing in Australia and New Zealand,* Melaleuca alternifolia*, using cold water extraction [28].

Tea tree oil is a mixture of terpene compounds, such as terpinen-4-ol, *γ*-terpinene,* p*-cymene, *α*-terpinene, and 1,8-cineole, which are largely responsible for tea tree oil's properties [29, 30]. TTO exhibits strong antiseptic activity, selective for pathogenic microorganisms, and is highly effective against oral cavity pathogens [31]. It has also been demonstrated that TTO has inhibitory properties against* Candida albicans* [32].

The study aims to assess the influence of toothpastes containing ethanolic extract of propolis and tea tree oil on oral hygiene in patients with partial dentures. The number of scientific reports on synergistic activity of these substances in the improvement of oral hygiene is insufficient [33, 34]. The aim of this study was to evaluate the influence of toothpaste with active substances of plant origin, such as ethanolic extract of propolis and tea tree oil, on microflora of the oral cavity and to evaluate the following indices: OHI-S, denture plaque index, mSBI, and API in patients with partial dentures.

## 2. Materials and Methods

This study was conducted between September 1, 2015, and March 1, 2016, in the Department of Prosthetic Dentistry, Akademickie Centrum Stomatologii i Medycyny Specjalistycznej (Bytom, Poland), and in Specialist Dental Clinic (Katowice, Poland), which provide comprehensive dental care for patients with removable dentures.

### 2.1. Toothpaste Preparations with Propolis and Tea Tree Oil

Two samples of toothpaste covered anonymously with blank white tag and marked only with letter A or C were compared: the studied toothpaste with the active ingredient (study group, A), 1,5% content of ethanol extract of Polish propolis, and with no active ingredients, placebo (control group, C). The second natural product valued for its properties was tea tree oil (TTO). It is obtained from the* Melaleuca alternifolia* plant. Two other species (*Melaleuca linariifolia* and* Melaleuca dissitiflora*) are also used to obtain essential oil, only if they contain more than 30% terpinen-4-ol in the essential oil. The major component of tea tree oil is terpinen-4-ol. Its content is 29–45%. The composition of active and placebo toothpaste had the following:

*Active Toothpaste*

aqua (up to 100% of weight),
glycerine (5–12%),
silica (10–14%),
sorbitol (10–20%),
hydroxyethyl cellulose (0.1–1%),
titanium dioxide (0.5–2%),
xanthan gum (0.3–1%),
*ethanolic extract of propolis (EEP) (1.0%),*
*tea tree oil (TTO) (1.0%),*
menthol oil (0.2%),
rosemary oil (0.1%).

*Placebo Toothpaste*

aqua,
glycerine,
silica,
sorbitol,
hydroxyethyl cellulose,
titanium dioxide,
xanthan gum.

### 2.2. Study Groups Inclusion and Extrusion Criteria

Subject qualification for the study was based on medical and dental history, interview, and review of clinical records. The exclusion and inclusion criteria from the investigation were as follows:

*Inclusion Criteria*

- written participation consent,
- age (40–85 years),
- patients lacking 5–8 teeth in the maxilla and mandible under prosthetic treatment,
- patients with remaining posterior occluding pairs with a minimum of two supporting zones according to Eichner index.

*Extrusion Criteria*

- lack of a written participation consent, painful masticatory dysfunction,
- edentulous patients or patients with residual dentition (Eichner subgroups C1–C3), patients with full dental arches or lacking 1-2 teeth in an arch,
- patients with retaining teeth mobility over +20 PTV tested with the Periotest instrument,
- patients with class V fillings or acrylic/ceramic crowns around the retaining teeth,
- patients with cancer,
- psychosomatic disorders,
- patients after trauma within the craniofacial region,
- pregnant and lactating females,
- patients suffering from asthma or atopic dermatitis, allergic to foods, drugs, or honey and its products, or having other allergy-related ailments.

The study was conducted with the prior approval from the Bioethics Committee of the Silesian Medical Chamber in Katowice, Poland, Resolution number 8/2015 of March 23, 2015.

### 2.3. Patients

37 patients aged 41–82 (57.0 ± 10.49) were qualified for the study, including 20 females (54%) and 17 males (46%). Patients were randomly divided into two subgroups. The study group consisted of 18 persons aged 41–79 (55.05 ± 11.09), who received the active toothpaste with propolis and tea tree oil, whereas the control group consisted of 19 patients aged 41–82 (66.79 ± 11.19), who were given the negative control toothpaste.

### 2.4. Clinical Examination Protocol

The studies were performed in two stages. The first stage consisted of a general medical and dental interview based on a survey and a form written particularly for this purpose. The study was extended by a survey concerning medical history and current diseases, allergies, taken medication, and environmental risk. Mobility of the retaining tooth was also measured with Periotest during the first visit, as a criterion qualifying the patient for the study. The values may be in the following ranges: −08 to +09, physiological teeth mobility; +10 to +19, perceptible teeth mobility; +20 to +29, moderate teeth mobility under investigator's pressure; +30 to +50, severe teeth mobility under pressure from the lips or tongue [35, 36]. Patients qualified for the study were evaluated to determine the value of selected oral hygiene indices included in the form, that is, OHI-S, denture plaque index, mSBI, and API. The measurement procedure according to the above-mentioned indices was carried out with use of a standard WHO periodontal probe. The measurement was based on the assessment of presence and size of plaque on selected tooth surfaces and bleeding from the interdental papilla.

Approximal Plaque Index (API), Lange and Ainamo, 1988: Presence of plaque is assessed only on occlusal surfaces. The presence of bacterial plaque is examined in quadrants 1 and 3 on occlusal surfaces on the side of the oral cavity proper and in quadrants 2 and 4 on occlusal surfaces on the side of the vestibule. The formula used to calculate the value of API [%] = the sum of interdental spaces with plaque/the sum of all examined surfaces × 100%. Index values were interpreted as follows: API 100–70%, bad oral hygiene; API 70–40%, average hygiene, improvement recommended; API 39–25%, quite good oral hygiene; API < 25%, optimal oral hygiene.

Simplified Oral Hygiene Index determines the amount of soft debris or calculus on the four buccal surfaces of the selected teeth: upper right first molar, upper right central incisor, upper left first molar, and lower central incisor, and on the lingual surfaces of the lower left first molar and lower right first molar.

Denture plaque index (Budtz-Jörgensen, 1978) provides the following description for the amount of denture plaque on the fitting surface: 0 = nonvisible, 1 = less than one-third covered, 2 = one-third to two-thirds covered, and 3 = more than two-thirds covered.

The periodontal status (gingival health) was evaluated with use of the modified Sulcus Bleeding Index (mSBI, Muhlemann-Son, 1971) and by recording only “bleeding presence” or “bleeding absence” for all existing teeth. The index describes the presence of localised bleeding in interdental spaces and interdental papilla. In quadrants 1 and 3 the vestibular side is examined, while in quadrants 2 and 4 the lingual one is examined.

After clinical examination acrylic partial dentures were made for all 37 patients. Both groups (study and control) were subjected to hygienisation of the oral cavity and instructed on how to properly brush their teeth with use of Fones circular technique. Fones circular technique consists of putting the toothbrush perpendicularly to slightly opened dental arches and performing circular movements. In the roll technique the toothbrush is placed on attached gingiva at the angle of 45° with the bristles pointed towards the root. Patients were also shown the proper denture hygiene. They were instructed to wash their dentures after every meal with use of a soft denture brush or with hands and hard soap. After brushing the dentures were to be carefully rinsed under running water and put in the oral cavity. Patients were advised not to use dentures on a 24/7 basis. Dentures were supposed to be removed for the night and kept in a dry place after prior washing. All patients were examined in three subsequent stages: preliminary qualification at baseline before hygiene procedure (1st assessment), a follow-up after 7 days (2nd assessment), and a follow-up 28 days after the initial examination (3rd assessment). Patients were examined for regression of lesions, frame of mind, and possible side effects, which was recorded on the control form. During each examination swabs were used for microbiological examination of oral cavity microflora. The microbial material for smear tests was collected from the floor of the mouth.

### 2.5. Bacterial Strains Isolation and Microbiological Investigation

Microbiological examinations were performed with use of classic methods employed in microbiological diagnostics. Material collected from the patients was cultured on proper growth media for proliferation and subsequent isolation of pure cultures. Aerobic bacteria were proliferated on Columbia agar solid medium with 5% sheep blood at 37°C. Anaerobic bacteria were proliferated on Schaedler K3 solid medium with 5% sheep blood at 37°C in anaerobic conditions obtained with Biomerieux GENbag anaer generators (Marcy-l'Étoile, France). Species identification was performed after isolation and proliferation of cultured microorganism strains with use of the following reagent sets: ENTEROtest 24N, NEFERMtest 24N, STREPTOtest 24, STAPHYtest, ANAEROtest 23, OXItest, PYRAtest, and TNW_lite 6.5 computer program for species identification of microorganisms (Erba-Lachema, Brno, Czech Republic). Also the following Biomerieux (Marcyl'Etoile, France) biochemical tests were used: Katalaza, Slidex Staph Kit, and API Candida. Performance and interpretation of results of the tests was carried out according to the manufacturer's recommendations with diagnostic reagent sets.

### 2.6. Statistical Analysis

The first stage of statistical analysis consisted of verification of the compatibility of the obtained index values and the number of bacteria with normal distribution with use of the Shapiro-Wilk test. Variables with normal distribution were presented with arithmetic mean and standard deviation, whereas nonparametric variables were presented with median and interquartile range. One-way analysis of variance (ANOVA) and Levene's test were used to compare the results of the study group with the control group for OHI, denture plaque index, and the number of bacteria. The comparison of results between groups was performed with the Tukey-Kramer method. Results obtained for OHI and denture plaque index were compared with Student's *t*-test for dependent and independent samples. For unrelated variables of API and SBI the results of study and control groups were compared with Mann–Whitney *U* test, Wilcoxon signed-rank test, and Friedman's ANOVA with Kendall's coefficient of concordance. The results were deemed to be statistically significant if *p* < 0.05.

## 3. Results

### 3.1. Oral Health Conditions

The general distribution range for API was presented in Table 1. Statistically significant differences between groups were reported 7 and 28 days after the initial examination (*p* < 0.05 and *p* < 0.001). Improvement of hygiene was reported both in the study group (A) and in the control group (C). It may be associated with the reduction of interproximal spaces in the lateral segments of maxilla and/or mandible. A significant improvement was only observed in the study group (A) (Table 1).

Another assessed index was the simplified Greene-Vermilion hygiene index. Statistically significant improvement of hygiene in the study group (A) was observed (Table 2). In the study group (A) the index was determined after 7 and 28 days of usage (*p* = 0.013221, *p* = 0.000468). No statistical significance was observed in the control group (C) (Table 2). Significant reduction was observed by comparing OHI 7 and 28.

**Figure 1:**
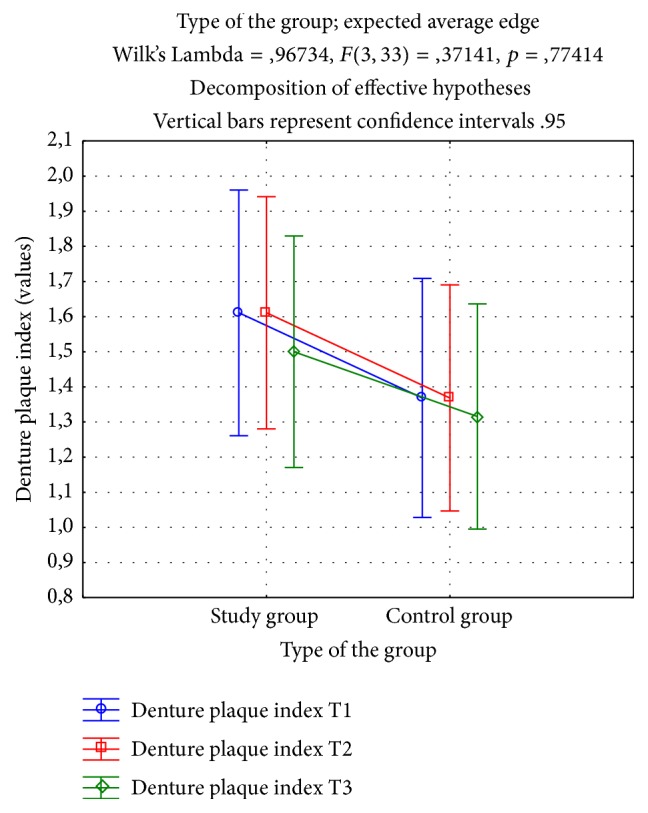
ANOVA Estimated Marginal Means for denture plaque index study and control group.

Based on the conducted analysis of denture plaque index values no significant reduction in the values was observed after the period of use of toothpastes in study (A) and control (C) groups compared to the output value (Figure 1).

Statistically significant reduction of gingival bleeding index after 7 and 28 days of the use of toothpastes compared to the gingival bleeding index at the beginning of the treatment was observed both in study (A) and in the control group (C) (Table 3). The study and control group did not differ in terms of the assessment of the SBI value upon initial examination. After 7 and 28 days of use the control group exhibited a significantly higher level of SBI in comparison with the study group (Table 3).

### 3.2. Microbiological Investigation

The following observations in study group (A) constituted by 18 patients were made: 77 microorganisms of 31 species were isolated in the first microbiological test. The second test (after 7 days of use of the toothpaste with EEP and TTO) resulted in the isolation of 78 microorganisms of 27 species. The third test after 4 weeks of use of the active toothpaste showed a decrease in the number of isolated microorganisms to 65, of 24 species (Table 4). The oral microflora diversity was reduced by the decrease in the number of cultured microorganism species. The following bacterial species were eliminated:* Actinomces viscocus, Actinomyces israelii, Atopobium parvulum, Bifidobacterium ovatus, Clostridium botulinum, Eubacterium saburreum, Lactobacillus acidophilus, Lactobacillus fermentum,* and* Propionibacterium granulosum,* classified as Gram(+) anaerobes. They are all part of the oral flora and, more specifically, dental biofilm bacteria.* Lactobacillus acidophilus* is a known pathogen responsible for the development of caries.* Actinomyces* spp. belong to pioneer bacteria responsible for the formation of plaque, while* A. israeli* may be an etiological factor for actinomycosis. The following species were also eliminated:* Bacteroides uniformis* and* Parabacteroides distasonis* classified as Gram(−) anaerobes;* Acinetobacter freundii*,* Burkholderia cepacia,* and* Neisseria sicca* (Gram(−) aerobe), as well as* Staphylococcus epidermidis* MSCNS (Gram(+) aerobe), which can cause opportunistic infections, including sepsis. The microflora gained the following species, Gram(+) anaerobes:* Atopobium minutum* and* Clostridium butyricum*, Gram(−) anaerobes:* Capnocytophaga ochracea* and* Prevotella melaninogenica*, Gram(+) aerobes:* Microccus* spp. and* Streptococcus oralis*, and Gram(−) aerobes:* Providencia rustigiani* and* Stenotrophomonas maltophilia*.* Micrococcus* spp. and* S. oralis* are classified as microorganisms which participate in plaque formation as one of the first.* Capnocytophaga ochracea* belongs to the green bacterial complex, associated with advanced periodontitis according to Socransky. In our study the number of the following bacteria declined:* Bifidobacterium adolescentis, Veillonella parvula, Streptococcus salivarius,* and* Streptococcus sanguinis*. They all belong to physiological bacterial flora and participate in the formation of dental biofilms. A reduction of* Candida albicans* was also observed (Table 5).* C. albicans* is often isolated from the oral cavity, although most frequently in patients using dentures, and does not always cause infection symptoms.

In the control group (C) constituted by 19 patients, 79 microorganisms of 25 species were isolated in the first microbiological test. The second test resulted in the isolation of 83 microorganisms of 28 species, while the third test after 4 weeks of use of the toothpaste without active ingredients resulted in the culture of 92 microorganisms of 26 species (Table 4). An increase of the number of cultured microorganisms and a slight increase in the number of species were observed. The following species were eliminated: Gram(+) anaerobes:* Clostridium sporogenes, Eubacterium saburreum,* and* Lactobacillus fermentum,* and Gram(−) anaerobes:* Bacteroides ovatus, Bacteroides uniformis, Fusobacterium mortiferum,* and* Prevotella oralis*, as well as Gram(+) aerobic bacterium* Staphylococcus aureus* MSSA. The microflora gained the following Gram(+) anaerobes:* Bifidobacterium dentium, Bifidobacterium ovatus, Clostridium chauvoei, Gemella morbillorum,* and* Propionibacterium propionicum. Bifidobacterium* spp. are probiotic bacteria dwelling in human intestine.* Gemella morbilorum* is a pathogen isolated from cysts which were not resolved after the second attempt of endodontic treatment. The flora also gained Gram(−) anaerobic bacterium* Veillonella parvula*, Gram(+) aerobic bacterium* Streptococcus mutans*, and Gram(−) aerobic bacterium* Neisseria sicca*.* Streptococcus* spp. participate in plaque formation, while* S. mutans* is a known etiological factor for caries.* V. parvula*, which belongs to the purple complex according to Socransky, is isolated in patients with healthy periodontium. The number of cultured* Candida albicans* also increased. As mentioned before, this fungus is often isolated in patients with removable dentures. The number of the following bacteria declined in the control group:* Blautia producta, Clostridium clostridiforme, Lactobacillus acidophilus, Capnocytophaga ochracea,* and* Streptococcus sanguinis*, and the number of the following bacteria increased:* Actinomyces israelii, Actinomyces naeslundii, Campylobacter gracilis, Streptococcus mitis, Streptococcus salivarium, Escherichia coli,* and* Neisseria subflava*, as well as fungi* Candida albicans* (Table 5).

### 3.3. Side Effects

No serious side effects have been reported. Some patients reported discoloration of their toothbrush.

## 4. Discussion

Natural extracts used in the tested toothpastes were intended to provide chemical assistance in the mechanical removal of plaque and thus improve oral hygiene. The first one was ethanolic extract of propolis. There are numerous studies confirming its strong antimicrobial, antiviral, antifungal, and antiphlogistic activity [37–39]. Propolis exhibits a wide spectrum of antimicrobial activities. Research conducted by Koru et al. demonstrated that propolis is more effective against Gram(+) aerobes than against Gram(−) aerobes [40]. In their study Feres et al. compared antimicrobial activity of 11% propolis and the popular 0.12% chlorhexidine against bacteria in saliva in healthy patients and in patients with periodontal diseases. Both substances exhibited statistically significant antimicrobial activity [41]. Antimicrobial activity of 10% propolis and 0.12% chlorhexidine against* S. mutans* and* L. acidofilus*, known pathogens responsible for the development of caries, was compared in 2011. Both proved to be effective [42]. Another study from 2013 compared 0.12% chlorhexidine with propolis at a much lower concentration, namely, 2%. Its antimicrobial activity against* S. mutans *and* L. acidofilus* was confirmed [43]. These studies proved the cariostatic properties of propolis, antiperiodontal pathogen activity, and efficiency at lower concentrations. In the present study we employed 1.5% ethanolic extract of propolis. Hitherto, there have been no studies confirming the antimicrobial effectiveness at the concentration of 1.5%. However, in the toothpaste tested it was combined with tea tree oil, which has similar properties. Thus, the concentration of EEP could be lowered. At the same time, lower concentration of EEP minimises the risk of side effects such as allergic reactions and burning sensation in the oral cavity [34]. An important aspect is the antiphlogistic properties of ethanolic extract of propolis [44]. Skaba et al. conducted an analysis of ethanolic extract of Brazilian propolis, in which they confirmed its antiphlogistic activity using three recognised methods. The same study included an assessment of the influence of toothpaste with 3% ethanolic extract of Brazilian propolis on oral hygiene with use of oral cavity indices: API, OHI, and SBI. Researchers collected also smears from patients in order to assess antimicrobial activity. Patients were divided into two groups. The study group (I) was using an active toothpaste (T) with propolis, while the control group (II) was using a toothpaste with no active substance (G). Patients were examined on the first, initial visit, second visit after a week, and third one after 4 weeks. After 4 weeks of using the T toothpaste there was a statistically significant decrease in OHI in both groups I and II, which may indicate that daily mechanical plaque removal, even without active substances, can be effective in healthy patients. API in group II did not change and patients were qualified for the group with average hygiene, whereas API in group I improved after 7 days and returned to baseline upon the last examination, patient with quite good hygiene. SBI improved in group I, but in a statistically insignificant way. It did not change in group II [45]. Study of toothpastes with 3% ethanolic extract of propolis in patients undergoing orthodontic treatment was conducted in 2013 and in 2016. Apart from the smears, it comprised also API according to Lange, Gingival Index (GI), and Orthodontic Plaque Index (OPI) upon first examination and after 35 days of using a toothpaste with propolis and CT gel. The control group consisted of patients using a toothpaste without propolis and CC gel. The first examination did not demonstrate any statistically significant differences in the assessed indices. Upon examination after 35 days there was a statistically significant decrease in the values of GI and OPI compared to the control group. No significant difference was shown in the decrease of API [46, 47].

*Melaleuca alternifolia* tea tree oil (TTO) is also a known antiseptic preparation with antimicrobial and antifungal activity. It has been successfully used for many centuries in dermatology, particularly in the treatment of acne vulgaris [48], accelerates wound healing, and has anticancer properties [49]. The basic composition of the oil is terpinen-4-ol, *γ*-terpinene, *α*-terpinene, 1,8-cineole, terpinolene, *ρ*-cymene, *α*-pinene, *α*-terpineol, aromadendrene, *δ*-cadinene, limonene, sabinene, globulol, and viridiflorol. They are terpene compounds with antiseptic and antiphlogistic activities which apart from dermatology can also be employed in dentistry, particularly in the maintenance of proper oral hygiene [50]. In 2003 Hammer et al. examined in vitro the antimicrobial activity of tea tree oil against 161 isolated oral pathogens and detected MIC and MBC for 15 of them. MIC and MBC values were lowest for* Porphyromonas* spp.,* Prevotella* spp., and* Veillonella* spp. and highest for isolated* Streptococcus* spp.,* Fusobacterium *spp., and* Lactobacillus* spp. Time necessary for the death of* Streptococcus mutans* and* Lactobacillus rhamnosus* in ≥0.5% concentration was 5 minutes. It was thus demonstrated that oral pathogens are susceptible to the activity of TTO [51].

Studies on toothpastes with natural extracts against three species of bacteria* S. mutans, P. aeruginosa,* and* E. faecalis* compared to a toothpaste without antimicrobial ingredients were conducted in 2015.* S. mutans* is a known etiological factor for caries, while* P. aeruginosa* and* E. faecalis* are pathogens isolated in periodontopathies and endodontic infections. 8 toothpastes with the following composition were used: sorbitol (I), tocopherol (II), mentha (III), cinnamon/mentha (IV), propolis/tea tree oil (V), mentha/açaí berry (VI), mentha/guarana (VII), and propolis (VIII). The control group was a toothpaste with no antimicrobial properties (group (IX), negative control), while group (X) (positive control) was a dental gel with triclosan and formaldehyde. The results demonstrated that* E. faecalis* exhibited resistance to the toothpaste with propolis and tea tree oil and susceptibility to the toothpaste with propolis only. The authors explain it with a different concentration of propolis in the toothpastes. Propolis/tea tree oil toothpaste exhibited the strongest antimicrobial activity against* S. mutans* compared to other toothpastes.* P. aeruginosa* proved to be resistant to all tested natural substances [52]. Some other researchers demonstrate alternative methods to reduce pathological oral microflora, by addition to resin composites on element ions with nanogold and nanosilver [53, 54].

In the present study there was an increase in the number of cultured* C. albicans* colonies in patients using both the active toothpaste and the placebo. Both active substances employed in the active toothpaste exhibit antifungal activity. However, it should be remembered that the group of examined patients was using acrylic partial dentures, which are an iatrogenic factor for* C. albicans* infection [55]. The use of acrylic partial dentures caused changes in the oral microflora. The acrylic material is a reservoir for* C. albicans*, whose amount depends on denture hygiene and its quality, particularly roughness [56, 57]. The 2011 study of bee products in comparison with fluconazole confirmed the antifungal activity of propolis and the possibility of its alternative use in antifungal therapy. It exhibits activity against* C. albicans, C. glabrata, C. krusei,* and* Trichosporon* spp. [58]. There are many reports on the antifungal activity of tea tree oil. The 2003 in vitro study examined the antifungal activity of particular components of the oil in concentrations between 0.5% and 2%. The results demonstrated that all components of the oil, apart from *β*-myrcene, exhibit antifungal activity [59]. The study from 2004 confirmed the hypothesis of the mechanism of action of tea tree oil against* C. albicans*,* C. glabrata,* and* S. cerevisiae*. The oil influences the properties and disrupts the functions of fungal cell membrane [60]. Study conducted by Sudjana et al. demonstrated that TTO inhibits adhesion of* C. albicans* to human cells and polystyrene and inhibits the formation of biofilm through reduction of surface tension [61].

## 5. Conclusions

1. Improvements in hygiene and the condition of periodontium were observed in patients using toothpastes with ethanolic extract of propolis and tea tree oil.
2. The toothpaste with ethanolic extract of propolis and tea tree oil had no influence on the reduction of denture plaque.
3. The studied toothpaste with active ingredients (ethanolic extract of propolis and tea tree oil) demonstrates the beneficial influence on microorganisms composition in oral microflora.

## Figures and Tables

**Table 1:** API ranges: assessment for study and control groups.

Oral hygiene assessment (interproximal spaces)						
	The assessment criteria	T1	T2	T3	Friedman's ANOVA test (*p*)/Kendall's coefficient of concordance	Wilcoxon signed-rank test *p*
Study group (A)	Optimal	11%	39%	56%	*p* = 0.0000/0.95894	(1) : (2) = 0.000438 (2) : (3) = 0.000293 (1) : (3) = 0.000196
	Quite good	15%	28%	44%		
	Average	53%	33%	0%		
	Bad	21%	0%	0%		

Control group (C)	Optimal	17%	21%	42%	*p* = 0.00228/0.32012	(1) : (2) = 0.231060 (2) : (3) = 0.064186 (1) : (3) = 0.000327
	Quite good	11%	37%	37%		
	Average	72%	42%	21%		
	Bad	0%	0%	0%		

Mann–Whitney “*U*” test (*p*)		0.784483	0.046554	0.001032	—	—

T1 (1): preliminary examination before hygiene procedure; T2 (2): examination after 7 days; T3 (3): 28 days after the initial examination.

**Table 2:** OHI ranges: assessment for study and control groups.

Oral hygiene assessment (interproximal spaces)											
	T1		Mean ± standard deviation	T2		Mean ± standard deviation	*p*	T3		Mean ± standard deviation	*p*
Study group (A)	0–0.5	55.5%	0.41 ± 0.25	0–0.5	66.6%	0.33 ± 0.23	0.013221	0–0.5	88.8%	0.20 ± 0.17	0.000468
	0.6–1	44.5%		0.6–1	34.4%			0.6–1	11.1%		
	1.1–2	0%		1.1–2	0%			1.1–2	0%		
	2.1–3	0%		2.1–3	0%			2.1–3	0%		

Control group (C)	0–0.5	58%	0.64 ± 0.50	0–0.5	63%	0.58 ± 0.30	0.352356	0–0.5	68.4%	0.57 ± 0.23	0.382331
	0.6–1	21%		0.6–1	21%			0.6–1	31.6%		
	1.1–2	16%		1.1–2	16%			1.1–2	0%		
	2.1–3	5%		0.006370	0.000002			2.1–3	0%		

Study (A) versus control (C) (*p*)	0.097301			0,006370				0,000002			

T1 (1): preliminary examination before hygiene procedure; T2 (2): examination after 7 days; T3 (3): 28 days after the initial examination.

**Table 3:** SBI ranges: assessment for study and control groups.

Sulcus Bleeding Index assessment						
	The assessment criteria	T1	T2	T3	Friedman's ANOVA test (*p*)/Kendall's coefficient of concordance	Wilcoxon signed-rank test *p*
Study group (A)	Normal gingiva SBI < 10%	50%	72.5%	100%	*p* = 0.00000/0.71010	(1) : (2): 0.003283 (2) : (3): 0.001474 (1) : (3): 0.000982
	Bleeding on probing	50%	27.5%	0%		

Control group (C)	Normal gingiva SBI < 10%	53%	65.5%	65.5%	*p* = 0.09760/0.12247	(1) : (2): 0.726768 (2) : (3): 0.286004 (1) : (3): 0.572082
	Bleeding on probing	47%	34.5%	34.5%		

Mann–Whitney *U* test (*p*)		0.346197	0.012713	0.000001		—

T1 (1): preliminary examination before hygiene procedure; T2 (2): examination after 7 days; T3 (3): 28 days after the initial examination.

**Table 4:** The microorganisms species isolated from patients, who used toothpaste with EEP and TTO (study group, A) or without EEP and TTO (control group, C).

Isolated microorganisms	Number of patients from study group (A)			Number of patients from control group (C)		
	Initial	After 7 days	After 28 days	Initial	After 7 days	After 28 days
Gram(+) anaerobic bacteria						
*Actinomyces viscosus *	1	1	0	0	0	0
*Actinomyces israelii *	1	1	0	2	3	3
*Actinomyces naeslundii*	1	2	1	3	7	7
*Anaerococcus prevotti *	0	0	0	0	1	0
*Atopobium minutum *	0	0	1	0	1	0
*Atopobium parvulum *	1	0	0	0	0	0
*Bifidobacterium adolesccentis *	2	1	1	1	0	1
*Bifidobacterium dentium *	1	0	1	0	1	3
*Bifidobacterium longum *	4	5	4	0	1	0
*Bifidobacterium breve *	0	0	0	5	2	5
*Bifidobacterium ovatus *	3	2	0	0	0	1
*Blautia producta *	1	0	2	2	0	1
*Clostridium barati *	1	1	1	0	2	0
*Clostridium botulinum biovar A *	1	1	0	0	0	0
*Clostridium butyricum*	0	0	1	0	0	0
*Clostridium chauvoei *	0	1	0	0	0	1
*Clostridium clostridiforme *	0	0	0	2	1	1
*Clostridium histolyticum *	0	1	0	0	0	0
*Clostridium perfringens *	0	1	0	1	1	1
*Clostridium sporogenes *	0	0	0	1	0	0
*Eubacterium saburreum *	1	1	0	1	0	0
*Gemella morbillorum *	0	0	0	0	0	1
*Lactobacillus acidophilus*	1	0	0	3	3	1
*Lactobacillus fermentum*	1	1	0	1	1	0
*Propionibacterium granulosum *	1	1	0	0	1	0
*Propionibacterium propionicum *	0	0	0	0	1	1

Gram(−) anaerobic bacteria						
*Bacteroides ovatus*	0	0	0	1	0	0
*Bacteroides uniformis *	1	0	0	1	0	0
*Campylobacter gracilis *	0	0	0	1	1	2
*Capnocytophaga ochracea *	0	1	1	3	2	1
*Fusobacterium nucleatum*	1	1	1	0	1	0
*Fusobacterium mortiferum *	0	0	0	1	0	0
*Mitsuokella multacida*	3	3	4	5	4	5
*Parabacteroides distasonis *	1	1	0	0	0	0
*Prevotella melaninogenica *	0	0	1	0	0	0
*Prevotella oralis *	0	0	0	1	0	0
*Veillonella parvula *	2	3	1	0	1	1

Gram(+) aerobic bacteria						
*Micrococcus* spp.	0	0	1	0	0	0
*Staphylococcus aureus MSSA *	1	0	2	1	0	0
*Staphylococcus epidermidis MSCNS*	1	0	0	0	0	0
*Streptococcus mitis *	7	10	7	6	7	9
*Streptococcus oralis *	0	1	1	0	0	0
*Streptococcus mutans*	0	0	0	0	0	2
*Streptococcus salivarius*	9	5	8	7	4	10
*Streptococcus sanguinis*	4	5	2	5	7	3

Gram(−) aerobic bacteria						
*Acinetobacter freundii*	1	0	0	0	0	0
*Burkholderia cepacia*	1	0	0	1	0	1
*Enterobacter amniogenus *	0	1	0	0	0	0
*Enterobacter cloacae *	0	1	0	0	0	0
*Enterobacter* spp.	0	0	0	0	1	0
*Escherichia coli *	0	0	0	1	1	2
*Neisseria sicca*	1	0	0	0	2	1
*Neisseria subflava *	15	18	16	18	16	19
*Providencia rustigiani *	0	0	1	0	0	0
*Pseudomonas* spp.	0	0	0	0	1	0
*Stenotrophomonas maltophilia *	0	0	1	0	0	0

Fungi						
*Candida albicans*	8	8	6	5	9	9

Number of microorganisms strains	**77**	**78**	**65**	**79**	**83**	**92**
Altogether:	**220**			**254**		

**Table 5:** The changes of oral microflora from patients, who used toothpaste with EEP and TTO (study group, A) or without EEP and TTO (control group, C).

Changes of microorganisms species after 28 days of study	Study group (A)	Control group (C)
Eliminated species	Gram(+) anaerobic bacteria	
	*Actinomyces viscosus* *Actinomyces israelii* *Atopobium parvulum Bifidobacterium ovatus Clostridium botulinum * *Eubacterium saburreum * *Lactobacillus acidophilus * *Lactobacillus fermentum * *Propionibacterium granulosum*	*Clostridium sporogenes* *Eubacterium saburreum* *Lactobacillus fermentum *
	Gram(−) anaerobic bacteria	
	*Bacteroides uniformis* *Parabacteroides dystasonis*	*Bacteroides ovatus* *Fusobacterium mortiferum* *Prevotella oralis*
	Gram(+) aerobic bacteria	
	*Staphylococcus epidermidis MSCNS*	*Staphylococcus aureus MSSA*
	Gram(−) aerobic bacteria	
	*Acinetobacter freundii* *Burkholderia cepacia* *Neisseria sicca *	—

Declined species	Gram(+) anaerobic bacteria	
	*Bifidobacterium adolescentis*	*Blautia producta* *Clostridium clostridiforme* *Lactobacillus acidophilus*
	Gram(−) anaerobic bacteria	
	*Veillonella parvula*	*Capnocytophaga ochracea*
	Gram(+) aerobic bacteria	
	*Streptococcus salivarius* *Streptococcus sanguinis*	*Streptococcus sanguinis*
	Fungi	
	*Candida albicans*	—

Gained species	Gram(+) anaerobic bacteria	
	*Atopobium minutum* *Clostridium butyricum*	*Bifidobacterium dentium Bifidobacterium ovatus * *Clostridium chauvoei * *Gemella morbillorum * *Propionibacterium propionicum*
	Gram(−) anaerobic bacteria	
	*Capnocytophaga ochracea Prevotella melaninogenica*	*Veillonella parvula*
	Gram(+) aerobic bacteria	
	*Micrococcus* spp. *Streptococcus oralis*	*Streptococcus mutans*
	Gram(−) aerobic bacteria	
	*Providiencia rustigiani* *Stenotrophomonas maltophilia*	*Neisseria sicca*

Increased species	Gram(+) anaerobic bacteria	
	*Blautia producta*	*Actinomyces israeli* *Actinomyces naeslundii*
	Gram(−) anaerobic bacteria	
	*Mitsuokella multiacida*	*Campylobacter gracilis*
	Gram(+) aerobic bacteria	
	*Staphylococcus aureus MSSA*	*Streptococcus mitis* *Streptococcus salivarius*
	Gram(−) aerobic bacteria	
	*Neisseria subflava*	*Neisseria subflava* *Escherichia coli*
	Fungi	
	—	*Candida albicans*
